# Deep learning-based quantitative analyses of spontaneous movements and their association with early neurological development in preterm infants

**DOI:** 10.1038/s41598-022-07139-x

**Published:** 2022-02-24

**Authors:** Hyun Iee Shin, Hyung-Ik Shin, Moon Suk Bang, Don-Kyu Kim, Seung Han Shin, Ee-Kyung Kim, Yoo-Jin Kim, Eun Sun Lee, Seul Gi Park, Hye Min Ji, Woo Hyung Lee

**Affiliations:** 1 Department of Rehabilitation Medicine, Chung-Ang University Hospital, Chung-Ang University College of Medicine, Seoul, Republic of Korea; 2 Department of Rehabilitation Medicine, Seoul National University Children’s Hospital, Seoul National University College of Medicine, 101 Daehak-ro, Jongno-gu, Seoul, 03080 Republic of Korea; 3 Department of Pediatrics, Seoul National University Children’s Hospital, Seoul National University College of Medicine, Seoul, Republic of Korea; 4 Department of Pediatrics, Chungbuk National University Hospital, Cheongju, Republic of Korea; 5 Department of Pediatrics, Chung-Ang University Hospital, Seoul, Republic of Korea

**Keywords:** Developmental biology, Neuroscience, Biomarkers

## Abstract

This study aimed to develop quantitative assessments of spontaneous movements in high-risk preterm infants based on a deep learning algorithm. Video images of spontaneous movements were recorded in very preterm infants at the term-equivalent age. The Hammersmith Infant Neurological Examination (HINE) was performed in infants at 4 months of corrected age. Joint positional data were extracted using a pretrained pose-estimation model. Complexity and similarity indices of joint angle and angular velocity in terms of sample entropy and Pearson correlation coefficient were compared between the infants with HINE < 60 and ≥ 60. Video images of spontaneous movements were recorded in 65 preterm infants at term-equivalent age. Complexity indices of joint angles and angular velocities differed between the infants with HINE < 60 and ≥ 60 and correlated positively with HINE scores in most of the joints at the upper and lower extremities (*p* < 0.05). Similarity indices between each joint angle or joint angular velocity did not differ between the two groups in most of the joints at the upper and lower extremities. Quantitative assessments of spontaneous movements in preterm infants are feasible using a deep learning algorithm and sample entropy. The results indicated that complexity indices of joint movements at both the upper and lower extremities can be potential candidates for detecting developmental outcomes in preterm infants.

## Introduction

Identifying high-risk indicators of cerebral palsy in preterm infants is crucial for early detection^1,2^. Early recognition of risks detectable in neonates can facilitate timely intervention and improve neurodevelopmental outcomes of high-risk infants^3,4^. Spontaneous movements in infants have been recognized as one of the most important predictors of cerebral palsy at the earliest possible age^5^. Spontaneous movements which begin in the early fetal period and last until the first 6 months post-term may reflect the integrity of the developing nervous system^6,7^. Currently, the General Movement Assessment (GMA) for evaluating spontaneous infantile movements is the validated tool for the early detection of cerebral palsy^5,8^ along with neonatal magnetic resonance imaging^9^ and the Hammersmith Infant Neurological Examination (HINE)^10^.

The GMA is a standardized motor assessment that uses video recordings of spontaneous infantile movements^6,11^. Previous studies have demonstrated that abnormal writhing movements and absent fidgety movements indicate an increased risk for adverse neurodevelopmental outcomes^1,12–14^. Thus, recent literature has recommended GMA for infants under 5 months of corrected age with newborn-detectable risks^1,15^. However, GMA for spontaneous movements in infants is conducted in a qualitative or semi-quantitative manner and may depend on skills and experience of the assessors. Certified assessors educated in high-quality training courses are necessary to conduct GMA, which may limit its clinical routine use^16^. To extend the availability of objective assessments of spontaneous infantile movement, it is important to quantitatively analyze the kinematics of the upper and lower extremities in preterm infants.

There have been previous attempts to analyze the spontaneous infantile movements based on automatic assessments. The automatic assessments can be helpful to reduce reliance on assessors, thereby decreasing human error. The sensing modalities of the automatic assessments in previous studies can be categorized into indirect sensing, where hardware is placed around the assessment environment (e.g. RGB cameras, 3D motion capture, and Microsoft Kinect) and direct sensing, where movements are captured using hardware attached to bodies (e.g. inertial sensors and magnet tracking systems)^17^. Particularly, the vision-based approaches using RGB cameras has been getting great interest with popularization of smartphones and recent advances in pose-estimation models based on deep learning algorithms. This approach can have several advantages compared to methods using direct sensing modalities including relative easiness to understand, high spatial resolution, high context information, non-intrusiveness, not-dependence on reflective markers or inertial sensors, and high availability^17,18^.

Previous researches using vision-based approaches have been steadily published for the past two decades^16,19^. To acquire data by tracking the location of the specific body structures, a motion tracking system using reflective markers^20,21^ were used and much simple methods using a single RGB camera have been adopted in recent studies^18,22–25^. This can be attributed to the progress of automatic pose-estimation techniques for two-dimensional image data and their disclosure as open source models including OpenPose^26^ and AlphaPose^27^. The previous studies have usually aimed to establish methods to quantify and classify spontaneous infantile movements^22–25,28^ or to develop prediction models to estimate developmental delay or cerebral palsy^15,18,20^. Although the classification or prediction performance of the previous models was relatively satisfactory, these models may provide information based on engineering techniques that can be unfamiliar or not be straightforward to clinicians to interpret and utilize for clinical decision-making. Clinicians may require more easily interpretable results based on quantitative assessments of important spatiotemporal variables.

Therefore, this study aimed to develop automatic standardized methods for quantitatively analyzing spontaneous movements in preterm infants and to analyze spontaneous movements according to early neurological development based on established quantitative assessments.

## Methods

### Participants

From March 2019 to January 2020, preterm infants with gestational age < 32 weeks or birth weight < 1500 g admitted to the neonatal intensive care units of two tertiary hospitals were enrolled in this study. Infants were excluded if they had genetic syndromes, major congenital malformations, or medically unstable conditions, such as requiring cardiovascular support, active sepsis, or any major surgery that could affect the spontaneous infantile movements. Informed consent was obtained from parents or legal guardians of all infants according to the institutional guidelines. After discharge, the participants entered the standard follow-up programs, including pediatric and neurologic evaluations and parental educational support. The study was approved by the institutional review boards of Seoul National University Hospital and Chung-Ang University Hospital. It was performed in accordance with all relevant guidelines and regulations.

### Clinical information

Clinical information of the infants at the perinatal and postnatal periods was obtained including infantile characteristics: sex, gestational age, postnatal age, birth weight, global score of HINE, corrected age at HINE, categories of GMA; neonatal morbidities: 1- and 5-min Apgar scores, bronchopulmonary dysplasia severity^29^, antenatal steroid use, periventricular leukomalacia, intraventricular hemorrhage, period of invasive ventilator use, patent ductus arteriosus, treatments of patent ductus arteriosus, retinopathy of prematurity, history of sepsis, and history of seizure; and maternal characteristics: maternal preeclampsia, gestational diabetes mellitus, multiple gestations, and maternal chorioamnionitis. Categories of GMA were determined by one certified expert who were blinded to the clinical history of the infants. Brain magnetic resonance imaging at term-equivalent age was performed in infants with birth weight < 1000 g, gestational age < 29 weeks, or those with severe intraventricular hemorrhage on brain ultrasonography.

### Video recordings of spontaneous movements

Video images of spontaneous movements in preterm infants were recorded according to the standard protocol of GMA at term-equivalent age (40 ± 1 weeks’ post-menstrual age)^8^. The instructions for making video recordings of spontaneous movements in this study were as follows: infants are (1) lying supine, naked, fully awakened, but without agitation, such as crying or fussing; (2) in a comfortable, quiet, and undisturbed condition at a neutral temperature; (3) free to move their bodies and all limbs including the fingers and toes, which are included in the recording scenes; and (4) recorded using a conventional or smartphone RGB camera^30,31^ for 3–5 min with at least 2 continuous min recorded by a research assistant with a certificate in occupational therapy or the parents. In cases of discharge from the hospital before the term-equivalent age, the parents were requested to record and transmit videos of their infants at home to the research team using their smartphone camera. The process of video recordings and transmission was explained to the parents or legal guardians before and after hospital discharge. If the recorded videos were considered unsuitable according to the instructions, recordings were conducted again within the period of term-equivalent age. The recorded videos were edited and combined into 3–5-min sequences.

### Automated movement recognition and kinematic analysis

**Figure 1:**
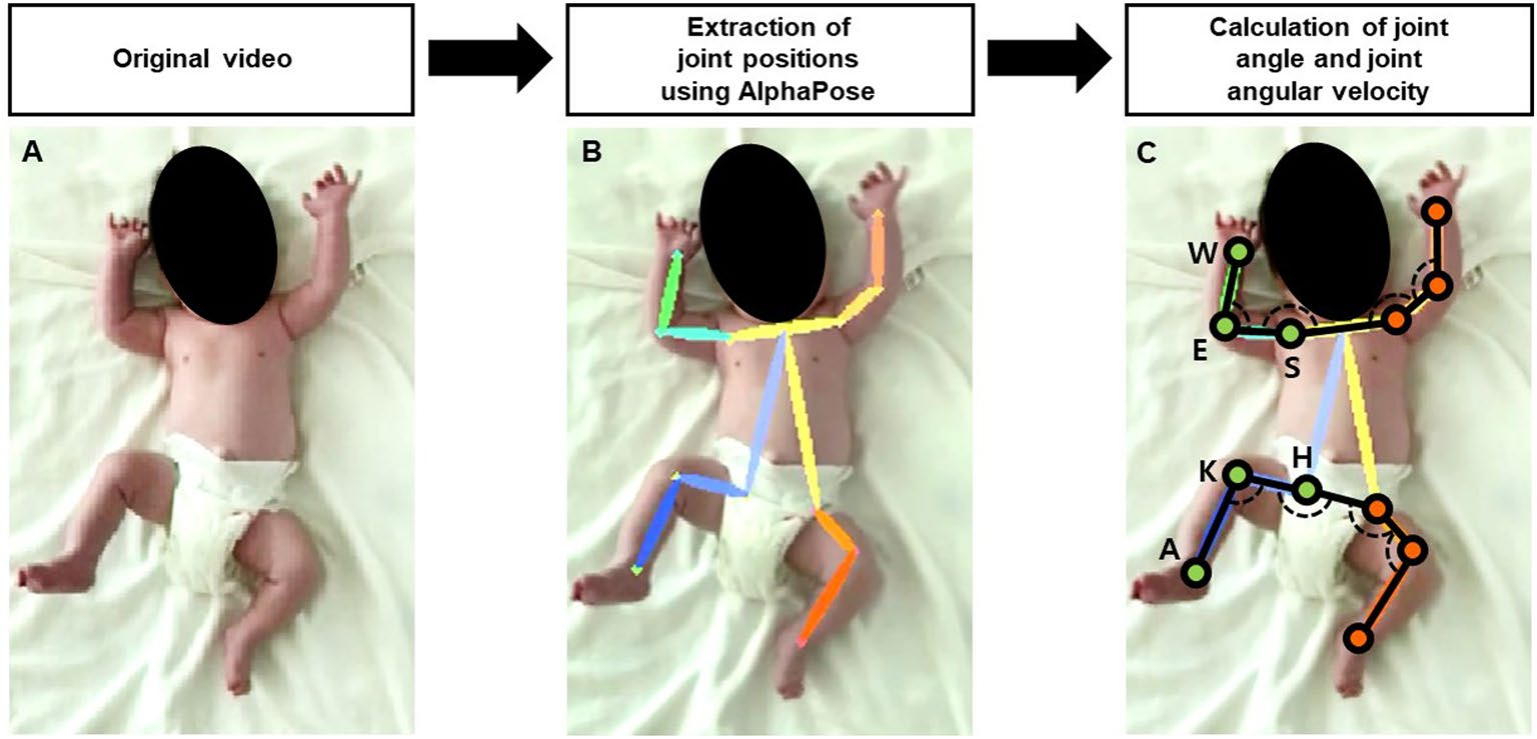
Overall processes of acquisition of joint angles and joint angular velocities at the upper and lower extremities from video images of spontaneous movements in preterm infants. *S* shoulder, *E* elbow, *W* wrist, *H* hip, *K* knee, *A* ankle.

The positional coordinates of twelve joints–the bilateral shoulders, elbows, wrists, hips, knees, and ankles–were automatically extracted from the recorded videos of spontaneous movements in the preterm infants using a pose-estimation algorithm, AlphaPose (Fig. 1A,B). This is a pretrained pose-estimation model developed based on a convolutional neural network architecture^27^. It can produce positional coordinates of each joint with confidence levels from 0 to 1 at each frame. The positional coordinates with confidence levels < 0.5 were regarded as measurement errors and eliminated from the positional data. Joint angles and joint angular velocities were determined as basic data in further kinematic analyses because these parameters are less susceptible to differences in camera distance from or angle of viewing of the infants compared to positional coordinates per se or the length between adjacent joints. The joint angle was defined as the angle formed by the positional coordinates of the corresponding joint and its two adjacent joints (Fig. 1C). The shoulder joint angle was defined as the angle made by the corresponding shoulder joint, contralateral shoulder joint, and ipsilateral elbow joint; the elbow joint angle is made by the corresponding elbow joint and the adjacent shoulder and wrist joints; the hip joint angle is made by the corresponding hip joint, contralateral hip joint, and ipsilateral knee joint; and the knee joint angle is made by the corresponding knee joint and the adjacent hip and ankle joints. The data of joint angles were acquired frame by frame and were interpolated with a locally weighted smoothing method for processing missing data and noise reduction. Joint angular velocities were approximated using the symmetric difference quotient as the sequence of the finite differences of the joint angles.

After preprocessing of time-series data of joint angles and joint angular velocities, the maximum, minimum, mean, and standard deviation values were calculated for each infant. Inter-limb synchronization and complexity during spontaneous movements were investigated with kinematic analysis since these are hallmarks of brain dysfunction and are associated with developmental disorders^32^. To quantify complexity of limb movements in preterm infants, this study adopted sample entropy (SE), a measure of the degree of signal regularity for time-series data as a complexity index^33^. Sample entropy is a measure of the degree of signal regularity, complexity, or ensemble orderliness for time-series data^34^. Reduced complexity in terms of sample entropy indicates that time-series data of joint angles and angular velocities are relatively more regular, less random, and less complex. Inter- and intra-limb synchronization of joint angles and joint angular velocities were quantified using Pearson correlation coefficients. These are measures of dependence between two paired data points with linear association and were used as a similarity index. It can reflect simultaneous changes of joint angles or joint angular velocities among bilateral upper and lower limbs: the joint angles and joint angular velocities of shoulders, elbows, hips, and knees at the right and left sides.

### Hammersmith infant neurological examination

The HINE was utilized to assess neurological outcomes in infants at 4 months of corrected age. The HINE was evaluated by an experienced physiatrist or physical therapist and was blinded to the infants’ medical histories. The HINE comprises five sections of examinations including assessments of cranial nerve function, posture, movements, tone, and reflexes and reactions with scores ranging from 0 to 78. The five sections were evaluated separately and then added to obtain the global scores. The global score at the corrected age of 3–4 months ranges from 62.5 to 69^35,36^. Global scores < 56 at 3 months of corrected age are considered highly indicative of cerebral palsy, and it is widely accepted that HINE scores of 40–60 at 3–6 months of corrected age indicate gross motor function classification system scores I–II^1,37^. Considering this information, the study participants were divided into the following two groups according to the HINE scores: HINE < 60 and ≥ 60.

### Statistical methods

The clinical characteristics were compared between infants with HINE < 60 and those with HINE ≥ 60 using Mann–Whitney *U* test for continuous variables and chi-squared test or Fisher’s exact test for categorical variables. Pearson correlation coefficients were obtained to analyze the correlations between HINE scores and complexity indices for joint angles and joint angular velocities. The complexity and similarity indices for joint angles and joint angular velocities were compared between the HINE < 60 and ≥ 60 groups using Student’s *t* test or Mann–Whitney *U* test. The significance level was set at *p* < 0.05. The statistical analyses were conducted using SPSS software (version 21; SPSS Inc, Chicago, IL). Complexity indices were calculated using R version 3.5.1 (The R Foundation, Vienna, Austria) with the pracma package.

## Results

### Clinical characteristics

In total, 65 infants who fulfilled the inclusion criteria were enrolled. Table 1 lists the clinical characteristics of 16 infants with HINE < 60 and 49 with HINE ≥ 60. There were significant differences between the two groups in the incidence of multiple gestations (*p* = 0.002) and patent ductus arteriosus (*p* = 0.040).

**Table 1:** Clinical characteristics.

Characteristics	Total (N = 65)	HINE < 60 (n = 16)	HINE ≥ 60 (n = 49)	*p*
**Infantile characteristics**				
Sex (female)	26 (40.0)	8 (50.0)	18 (36.7)	0.347
Gestational age (weeks:days)	28:5 (3:0)	28:5 (3:2)	28:5 (2:6)	0.996
Postnatal age (weeks:days) at video recordings	11:3 (3:0)	11:3 (3:2)	11:4 (3:0)	0.854
Birth weight (g)	1093.9 (358.3)	1128.7 (421.6)	1080.3 (340.7)	0.880
Global score of HINE	64.7 (9.5)	51.1 (5.9)	69.2 (5.3)	–
Corrected age at HINE (months:weeks)	3:3 (0:5)	3:3 (0:5)	3:3 (0:5)	0.894
Categories of General movement assessments				0.071
Poor repertoire	12 (18.5)	6 (37.5)	6 (12.2)	
Cramped synchronized	12 (18.5)	3 (18.8)	9 (18.4)	
Chaotic	5 (7.7)	2 (12.5)	3 (6.1)	
**Neonatal morbidities**				
1-Min Apgar score^a^	4.2 (2.1)	3.6 (2.3)	4.4 (2.0)	0.136
5-Min Apgar score^a^	6.5 (2.2)	6.1 (2.3)	6.6 (2.1)	0.476
Bronchopulmonary dysplasia (moderate to severe)	24 (57.1)	5 (35.7)	19 (67.9)	0.767
Antenatal steroid^a^	31 (48.4)	8 (50.0)	23 (47.9)	0.885
Periventricular leukomalacia	11 (16.9)	5 (31.3)	6 (12.2)	0.078
Intraventricular hemorrhage (grade III-IV)	9 (13.8)	4 (25.0)	5 (10.2)	0.207
Invasive ventilator use (days)	17 (24)	24 (33)	15 (21)	0.320
Patent ductus arteriosus	37 (56.9)	13 (81.3)	24 (49.0)	0.040*
Treatments of patent ductus arteriosus				0.073
Pharmacological closure	12 (18.5)	6 (37.5)	6 (12.2)	
Surgical closure	13 (20.0)	2 (12.5)	11 (22.4)	
Retinopathy of prematurity (stage 3–5)	7 (10.8)	1 (6.3)	6 (12.2)	0.671
Sepsis	13 (20.0)	4 (25.0)	9 (18.4)	0.720
Seizure	6 (9.2)	3 (18.8)	3 (6.1)	0.154
**Maternal characteristics**				
Maternal preeclampsia	10 (15.4)	3 (18.8)	7 (14.3)	0.698
Gestational diabetes mellitus	8 (12.3)	2 (12.5)	6 (12.2)	1.000
Multiple gestations	41 (63.1)	5 (31.3)	36 (73.5)	0.002*
Maternal chorioamnionitis	26 (40.0)	6 (37.5)	20 (40.8)	0.814

Values are presented as mean (standard deviation) or number of participants.

*HINE* Hammersmith infant neurological examination.

*p < 0.05.

^a^One patient was not included in the analysis due to missing data.

### Descriptive statistics of spontaneous movements

**Figure 2:**
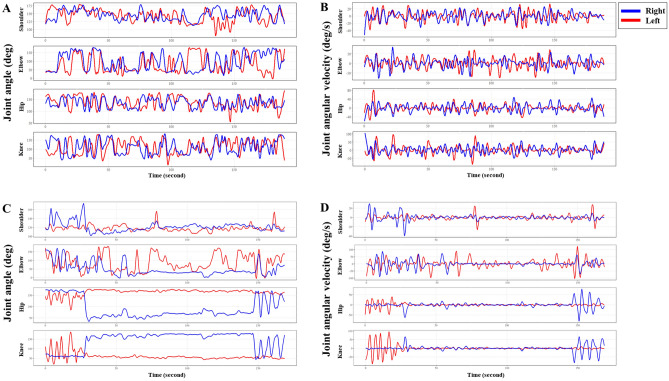
Illustrations of joint angles and joint angular velocities at the bilateral shoulders, elbows, hips, and knees in two preterm infants with Hammersmith Infant Neurological Examination (HINE) ≥ 60 (**A**,**B**) and those with HINE < 60 (**C**,**D**).

Illustrations of joint angles and joint angular velocities at the bilateral shoulders, elbows, hips, and knees are shown in Fig. 2. For the upper and lower extremities, descriptive statistics of joint angles and joint angular velocities including maximum, mean, minimum, and standard deviation were analyzed (Supplementary Tables S1 and S2). The maximum and mean of joint angles at the right elbow (156.85 [26.97] vs. 174.88 [14.42], *p* = 0.009; 70.01 [29.66] vs. 90.91 [27.1], *p* = 0.011) and standard deviation of joint angles at the right and left shoulders (13.31 [2.96] vs. 15.38 [3.19], *p* = 0.021; 14.06 [8.63] vs. 16.57 [4.25], *p* = 0.009) differed significantly between the infants with HINE < 60 and ≥ 60. The maximum, minimum, and standard deviation of joint angular velocities at the right shoulder differed significantly between the two groups (34.55 [16.41] vs. 48.20 [24.26], *p* = 0.027; − 35.08 [16.39] vs. − 46.72 [22.79], *p* = 0.048; 7.81 [3.65] vs. 10.83 [4.87], *p* = 0.017). The mean values of joint angular velocities at the right hip and knee differed significantly between the two groups (− 0.14 [0.15] vs. 0.03 [0.17], *p* = 0.001; 0.16 [0.31] vs. − 0.05 [0.26], *p* = 0.010). The standard deviation in joint angular velocities at the right elbow, right knee, and left knee differed significantly between the two groups (17.53 [8.94] vs. 24.62 [9.31], *p* = 0.017; 14.97 [8.1] vs. 20.79 [8.62], *p* = 0.022; 14.98 [7.78] vs. 19.46 [7.75], *p* = 0.049).

### Complexity and similarity indices of spontaneous movements

The complexity indices of joint angles and angular velocities had significant positive correlations with HINE scores in most of the joints at the upper and lower extremities within the range of 0.2–0.4 (Table 2). The complexity indices of joint angles and angular velocities differed significantly between the infants with HINE < 60 and ≥ 60 in most of the joints at the upper and lower extremities (Table 3).

**Table 2:** Results of correlation coefficients between global scores of Hammersmith Infant Neurological Examination and complexity indices for joint angles and joint angular velocities (N = 65).

	Correlation coefficients	*p*
**Joint angle**		
Right shoulder	0.288	0.020*
Left shoulder	0.277	0.026*
Right elbow	0.206	0.099
Left elbow	0.234	0.060
Right hip	0.227	0.069
Left hip	0.312	0.012*
Right knee	0.246	0.048*
Left knee	0.315	0.011*
**Joint angular velocity**		
Right shoulder	0.267	0.032*
Left shoulder	0.279	0.024*
Right elbow	0.244	0.051
Left elbow	0.264	0.034*
Right hip	0.281	0.023*
Left hip	0.370	0.002*
Right knee	0.228	0.068
Left knee	0.386	0.001*

*p < 0.05.

**Table 3:** Comparison results of complexity indices for joint angle and joint angular velocity between preterm infants with Hammersmith Infant Neurological Examination (HINE) < 60 and those with HINE ≥ 60.

	HINE < 60 (n = 16)	HINE ≥ 60 (n = 49)	*p*
**Joint angle**			
Right shoulder	0.050 (0.031)	0.074 (0.038)	0.025*
Left shoulder	0.056 (0.036)	0.074 (0.038)	0.083
Right elbow	0.042 (0.028)	0.064 (0.038)	0.048*
Left elbow	0.049 (0.035)	0.065 (0.040)	0.106
Right hip	0.049 (0.030)	0.068 (0.039)	0.053
Left hip	0.046 (0.025)	0.069 (0.030)	0.009*
Right knee	0.043 (0.030)	0.062 (0.033)	0.014*
Left knee	0.043 (0.022)	0.064 (0.030)	0.008*
**Joint angular velocity**			
Right shoulder	0.122 (0.068)	0.166 (0.072)	0.041*
Left shoulder	0.121 (0.069)	0.168 (0.069)	0.031*
Right elbow	0.100 (0.068)	0.148 (0.078)	0.027*
Left elbow	0.113 (0.078)	0.154 (0.081)	0.057
Right hip	0.122 (0.063)	0.160 (0.063)	0.029*
Left hip	0.112 (0.045)	0.163 (0.060)	0.003*
Right knee	0.108 (0.065)	0.140 (0.057)	0.023*
Left knee	0.095 (0.052)	0.146 (0.062)	0.003*

Values are presented as mean (standard deviation).

*p < 0.05.

Joint angles and angular velocities indicated very weak or weak correlations in most of the joints at the upper and lower extremities, except for the ipsilateral hip and knee joints, which showed strong or very strong correlations (Supplementary Tables S3 and S4). The correlation coefficients of joint angles between the right and left hips (0.06 [0.35] vs. 0.27 [0.26], *p* = 0.016), left shoulder and elbow (0.23 [0.27] vs. 0.08 [0.34], *p* = 0.029), and left shoulder and hip (0.16 [0.23] vs. 0.00 [0.19], *p* = 0.010) differed significantly between the infants with HINE < 60 and ≥ 60. There was no significant difference in the correlation coefficients among joint angular velocities between the two groups.

## Discussion

The current study demonstrated that the complexity of term-equivalent spontaneous movements in preterm infants quantitatively analyzed using a deep learning algorithm is associated with early neurological development assessed by HINE at 4 months of corrected age^38,39^. The complexity indices of both joint angles and joint angular velocities were different between the very preterm infants with HINE < 60 and ≥ 60, and showed positive correlations with the HINE scores in most joints of the upper and lower extremities. The similarity indices among each joint angle or among each joint angular velocity did not differ between the infants with HINE < 60 and ≥ 60 in most of the joints at the upper and lower extremities.

In this study, reduced complexity of spontaneous movements was significant or demonstrated a trend towards significance for all of the upper and lower extremities in the preterm infants with early neurological development. Global scores of HINE at 3–6 months of corrected age are significantly associated with levels of Gross Motor Function Classification System in cerebral palsy and cognition at 2 years of age in previous studies^38,39^. The complexity indices of both joint angles and joint angular velocities showed a significant positive linear relationship with the global HINE scores even though the strength of relationships was weak. These results are overall consistent with those of previous reports that describe decreased complexity as one of the important findings in infants at a high risk of cerebral palsy^36,40,41^. Hypothetically, spontaneous movements are endogenously generated by the central pattern generator network in the spinal cord and brainstem^6^. Its activity may result in simple body movements but can be modulated by the activity of the supraspinal structures (e.g. subplate and cortical plate) during brain development; this may induce movement complexity^40^. One of the characteristics in deviant patterns of the spontaneous movements is a lack of variability; this can result from injuries or dysfunction of the subplate or cortical plate and/or its connective fibres^41^.

Complexity, which comprises spatial and temporal variability, was investigated to objectively measure spontaneous movements in preterm infants since reduced complexity is strongly associated with cerebral palsy^40,41^. SE was adopted as a key index to measure complexity quantitatively. Generally, reduced complexity indicates fetal or neonatal compromise and has been regarded as one of the important characteristics to distinguish normal and abnormal spontaneous movements during the writhing period^42,43^. A previous study demonstrated that SE of time-series kinematic data acquired from infants with inertial sensors attached to the lower extremities is associated with a risk of developmental delay^33^. In that study, SE of the lower-limb movements was significantly decreased in the infants at risk of developmental delay, which is consistent with the results of the current study. Conversely, similarity in terms of the Pearson correlation coefficients did not correspond with the results of complexity. It may be assumed that the similarity of the inter- or intra-limb movements reflects the characteristics of cramped-synchronized movements, but the similarity index, in terms of Pearson’s correlation coefficients, may be insufficient to discriminate infants according to the early neurological development. It is necessary to plan future studies that reveal the quantitative features of each type of spontaneous movements according to GMA.

Automatic computer-based analyses of spontaneous infantile movements have been explored for clinical use in numerous previous studies^16,17^. One of the main approaches applied is a two-dimensional video-based approach. This has been strengthened by the rapid progress of computer vision technologies, such as deep learning algorithms, and the popularization of smartphone cameras^19^. In the current study, AlphaPose, an open-source pose-estimation model that demonstrates state-of-the-art performance, was used to extract the positional coordinates of each joint at the upper and lower extremities^27^. The acquisition of positional coordinates was conducted automatically using AlphaPose, facilitating the efficient performance of kinematic analyses of spontaneous infantile movements. The pose-estimation model provides automatic acquisition of spatiotemporal data on the trunk, arms, and legs of infants without labour-intensive and time-consuming manual marking. This study determined the kinematic parameters as joint angles and joint angular velocities, rather than as the raw data of the positional coordinates. Joint angles were considered beneficial since they can be less affected by the camera’s height, tilt/roll angles, and directions compared to positional coordinates and be straightforward for clinicians to analyze^44–46^.

This study has several limitations. First, in this study, a neurological outcome in infants was the global score of HINE at 4 months of corrected age. Even though a low global score of HINE at an early stage is suggestive of developmental delay or cerebral palsy, future studies are necessary to identify the long-term longitudinal association between complexity indices of spontaneous infantile movements and poor neurological outcomes. Second, the sample size of this study was relatively small and a further study is warranted with a large population of preterm infants. Third, kinematic analyses were performed on the two-dimensional video images, not on the three-dimensional image analyses of spontaneous movements in preterm infants. To obtain easy access to video images of the infants after hospital discharge, this study adopted the image acquisition instruments of conventional or smartphone RGB cameras.

In conclusion, quantitative assessments of spontaneous movements in preterm infants using a deep learning algorithm and sample entropy are feasible. Complexity indices in terms of SE of joint angles and joint angular velocities at both the upper and lower extremities are associated with early neurological development. This study indicates that complexity indices of spontaneous movements can be potential candidates to detect cerebral palsy in high-risk infants. Further studies are warranted with larger cohorts of preterm infants and a focus on developing automatic computer-based models using complexity indices for predicting developmental outcomes in clinical practice.

## Supplementary Information

Supplementary Information 1.

Supplementary Information 2.

Supplementary Information 3.

Supplementary Information 4.

## Data Availability

The data that support the findings of this study are available from the corresponding author upon reasonable request.

## References

[1] Novak I (2017). Early, accurate diagnosis and early intervention in cerebral palsy: Advances in diagnosis and treatment. JAMA Pediatr..

[2] Einspieler C (2019). Cerebral palsy: Early markers of clinical phenotype and functional outcome. J. Clin. Med..

[3] King AR (2021). Early detection of cerebral palsy in high-risk infants: Translation of evidence into practice in an Australian hospital. J. Paediatr. Child Health.

[4] Morgan C (2019). The pooled diagnostic accuracy of neuroimaging, general movements, and neurological examination for diagnosing cerebral palsy early in high-risk infants: A case control study. J. Clin. Med..

[5] Prechtl HF (1997). An early marker for neurological deficits after perinatal brain lesions. Lancet.

[6] Einspieler C, Prechtl HF (2005). Prechtl's assessment of general movements: a diagnostic tool for the functional assessment of the young nervous system. Ment. Retard. Dev. Disabil. Res. Rev..

[7] Einspieler, C., Prayer, D. & Marschik, P. B. Fetal movements: the origin of human behaviour. *Dev. Med. Child Neurol.* (2021). 10.1111/dmcn.14918 33973235

[8] Einspieler, C. *Prechtl's method on the qualitative assessment of general movements in preterm, term and young infants*. (Mac Keith Press London, 2004). 10.1016/s0378-3782(97)00092-3 9467693

[9] Ashwal S (2004). Practice parameter: diagnostic assessment of the child with cerebral palsy: report of the Quality Standards Subcommittee of the American Academy of Neurology and the Practice Committee of the Child Neurology Society. Neurology.

[10] Romeo DM, Ricci D, Brogna C, Mercuri E (2016). Use of the Hammersmith Infant Neurological Examination in infants with cerebral palsy: a critical review of the literature. Dev. Med. Child Neurol..

[11] Bosanquet M, Copeland L, Ware R, Boyd R (2013). A systematic review of tests to predict cerebral palsy in young children. Dev. Med. Child Neurol..

[12] Einspieler C, Peharz R, Marschik PB (2016). Fidgety movements–tiny in appearance, but huge in impact. J. Pediatr. (Rio J).

[13] Einspieler C (2014). Highlighting the first 5 months of life: general movements in infants later diagnosed with autism spectrum disorder or Rett syndrome. Res. Autism Spectr. Disord..

[14] Herrero D (2017). The motor repertoire in 3-to 5-month old infants with Down syndrome. Res. Dev. Disabil..

[15] Adde L (2010). Early prediction of cerebral palsy by computer-based video analysis of general movements: A feasibility study. Dev. Med. Child Neurol..

[16] Silva, N. *et al.* The future of General Movement Assessment: The role of computer vision and machine learning–A scoping review. *Res. Dev. Disabil.* **110**, 103854 (2021). 10.1016/j.ridd.2021.103854 PMC7910279 33571849

[17] Marcroft C, Khan A, Embleton ND, Trenell M, Plötz T (2015). Movement recognition technology as a method of assessing spontaneous general movements in high risk infants. Front. Neurol..

[18] Ihlen EA (2020). Machine learning of infant spontaneous movements for the early prediction of cerebral palsy: A multi-site cohort study. J. Clin. Med..

[19] Irshad MT, Nisar MA, Gouverneur P, Rapp M, Grzegorzek M (2020). Ai approaches towards Prechtl’s assessment of general movements: A systematic literature review. Sensors.

[20] Kanemaru N (2013). Specific characteristics of spontaneous movements in preterm infants at term age are associated with developmental delays at age 3 years. Dev. Med. Child Neurol..

[21] Meinecke L (2006). Movement analysis in the early detection of newborns at risk for developing spasticity due to infantile cerebral palsy. Hum. Mov. Sci..

[22] Marchi V (2019). Automated pose estimation captures key aspects of General Movements at eight to 17 weeks from conventional videos. Acta Paediatr..

[23] Baccinelli W (2020). Movidea: A software package for automatic video analysis of movements in infants at risk for neurodevelopmental disorders. Brain Sci..

[24] Tsuji T (2020). Markerless measurement and evaluation of general movements in infants. Sci. Rep..

[25] Reich S (2021). Novel AI driven approach to classify infant motor functions. Sci. Rep..

[26] Cao, Z., Simon, T., Wei, S. E., & Sheikh, Y. Realtime multi-person 2D pose estimation using part affinity fields. in *Proceedings of the IEEE conference on computer vision and pattern recognition.* 7291–7299 (2017)

[27] Fang, H.-S., Xie, S., Tai, Y. W., & Lu, C. Rmpe: Regional multi-person pose estimation. in *Proceedings of the IEEE international conference on computer vision* 2334–2343 (2017).

[28] McCay KD (2020). Abnormal infant movements classification with deep learning on pose-based features. IEEE Access.

[29] Malavolti, A. M. *et al.* Bronchopulmonary dysplasia—impact of severity and timing of diagnosis on neurodevelopment of preterm infants: A retrospective cohort study. *BMJ Paediatr. Open* **2** (2018). 10.1136/bmjpo-2017-000165 PMC5842992 29637181

[30] Marschik PB (2017). A novel way to measure and predict development: A heuristic approach to facilitate the early detection of neurodevelopmental disorders. Curr. Neurol. Neurosci. Rep..

[31] Spittle, A. *et al.* The Baby Moves prospective cohort study protocol: using a smartphone application with the General Movements Assessment to predict neurodevelopmental outcomes at age 2 years for extremely preterm or extremely low birthweight infants. *BMJ Open* **6**, e013446 (2016). 10.1136/bmjopen-2016-013446 PMC5073614 27697883

[32] Hadders-Algra M (2004). General movements: A window for early identification of children at high risk for developmental disorders. J. Pediatr..

[33] Smith BA, Vanderbilt DL, Applequist B, Kyvelidou A (2017). Sample entropy identifies differences in spontaneous leg movement behavior between infants with typical development and infants at risk of developmental delay. Technologies.

[34] Lake DE, Richman JS, Griffin MP, Moorman JR (2002). Sample entropy analysis of neonatal heart rate variability. Am. J. Physiol. Regul. Integr. Comp. Physiol..

[35] Haataja L (2003). Application of a scorable neurologic examination in healthy term infants aged 3–8 months. J. Pediatr..

[36] Romeo DM (2016). Early psychomotor development of low-risk preterm infants: Influence of gestational age and gender. Eur. J. Paediatr. Neurol..

[37] Romeo DM, Cioni M, Palermo F, Cilauro S, Romeo MG (2013). Neurological assessment in infants discharged from a neonatal intensive care unit. Eur. J. Paediatr. Neurol..

[38] Romeo DM (2008). Neuromotor development in infants with cerebral palsy investigated by the Hammersmith Infant Neurological Examination during the first year of age. Eur. J. Paediatr. Neurol..

[39] Romeo DM (2021). Hammersmith Infant Neurological Examination for infants born preterm: Predicting outcomes other than cerebral palsy. Dev. Med. Child Neurol..

[40] Hadders-Algra M (2018). Neural substrate and clinical significance of general movements: an update. Dev. Med. Child Neurol..

[41] Hadders-Algra M (2007). Putative neural substrate of normal and abnormal general movements. Neurosci. Biobehav. Rev..

[42] Einspieler C, Bos AF, Libertus ME, Marschik PB (2016). The general movement assessment helps us to identify preterm infants at risk for cognitive dysfunction. Front. Psychol..

[43] Prechtl HF (1997). State of the art of a new functional assessment of the young nervous system: An early predictor of cerebral palsy. Early Hum. Dev..

[44] Moccia S, Migliorelli L, Carnielli V, Frontoni E (2019). Preterm infants’ pose estimation with spatio-temporal features. IEEE Trans. Biomed. Eng..

[45] Yahya, M. *et al.* Real time elbow angle estimation using single RGB camera. *arXiv preprint arXiv:1808.07017* (2018).

[46] Kim HY (2015). The examination of reliability of lower limb joint angles with free software ImageJ. J. Ergon. Soc. Korea.

