# Supplementary material for: Deep learning-based quantitative analyses of spontaneous movements and their association with early neurological development in preterm infants
Source: Sci Rep. 2022 Feb 24;12:3138. doi: 10.1038/s41598-022-07139-x (PMC8873498; doi:10.1038/s41598-022-07139-x)
Supplement: Supplementary file 1 — Supplementary Information 1. [file 41598_2022_7139_MOESM1_ESM.docx]

**Supplementary Table S1.** Comparison of maximal, mean, minimal, and standard deviation (SD) values of joint angles between preterm infants with Hammersmith Infant Neurological Examination (HINE)<60 and those with HINE≥60

|  |  | HINE<60 (n=16) | HINE≥60 (n=49) | *p* |
| --- | --- | --- | --- | --- |
| Right shoulder | Max | 172.16 (8.03) | 176.33 (4.35) | 0.097 |
|  | Mean | 135.22 (19.35) | 140.59 (10.01) | 0.301 |
|  | Min | 97.01 (22.31) | 93.24 (29.08) | 0.976 |
|  | SD | 13.31 (2.96) | 15.38 (3.19) | 0.021**^a^** |
| Left shoulder | Max | 170.22 (10.57) | 176.49 (2.56) | 0.074 |
|  | Mean | 131.60 (15.43) | 139.5 (10.35) | 0.070 |
|  | Min | 93.95 (34.58) | 90.43 (31.42) | 0.738 |
|  | SD | 14.06 (8.63) | 16.57 (4.25) | 0.009**^a^** |
| Right elbow | Max | 156.85 (26.97) | 174.88 (14.42) | 0.009**^a^** |
|  | Mean | 70.01 (29.66) | 90.91 (27.10) | 0.011**^a^** |
|  | Min | 6.72 (11.9) | 10.6 (13.85) | 0.411 |
|  | SD | 34.22 (11.15) | 38.41 (7.83v | 0.179 |
| Left elbow | Max | 164.31 (27.13) | 172.4 (13.93) | 0.217 |
|  | Mean | 74.94 (31.19) | 90.81 (27.39) | 0.056 |
|  | Min | 4.97 (10.65) | 9.86 (17.71) | 0.394 |
|  | SD | 37.22 (12.04) | 38.46 (9.07) | 0.663 |
| Right hip | Max | 173.85 (8.57) | 174.71 (6.95) | 0.522 |
|  | Mean | 125.86 (20.58) | 128.87 (15.28) | 0.534 |
|  | Min | 77.25 (23.90) | 73.86 (21.48) | 0.605 |
|  | SD | 21.30 (7.94) | 21.72 (5.13) | 0.803 |
| Left hip | Max | 171.91 (9.43) | 174.04 (10.77) | 0.385 |
|  | Mean | 131.20 (31.80) | 130.92 (15.10) | 0.322 |
|  | Min | 72.13 (37.24) | 77.37 (23.95) | 0.843 |
|  | SD | 18.14 (4.88) | 20.13 (6.39) | 0.223 |
| Right knee | Max | 172.48 (12.92) | 178.05 (5.53) | 0.161 |
|  | Mean | 118.42 (25.09) | 116.11 (23.44) | 0.737 |
|  | Min | 44.87 (22.67) | 40.47 (20.26) | 0.467 |
|  | SD | 29.24 (8.36) | 32.23 (7.01) | 0.162 |
| Left knee | Max | 173.02 (23.20) | 177.13 (6.53) | 0.648 |
|  | Mean | 100.77 (33.74) | 114.63 (22.88) | 0.141 |
|  | Min | 37.63 (25.64) | 47.98 (21.20) | 0.113 |
|  | SD | 25.98 (9.61) | 30.35 (6.85) | 0.050 |

Values are presented as mean (standard deviation). **^a^***p*<0.05.
