# Supplementary material for: Deep learning-based quantitative analyses of spontaneous movements and their association with early neurological development in preterm infants
Source: Sci Rep. 2022 Feb 24;12:3138. doi: 10.1038/s41598-022-07139-x (PMC8873498; doi:10.1038/s41598-022-07139-x)
Supplement: Supplementary file 2 — Supplementary Information 2. [file 41598_2022_7139_MOESM2_ESM.docx]

**Supplementary Table S2.** Comparison of maximal, mean, minimal, and standard deviation (SD) values of joint angular velocities between preterm infants with Hammersmith Infant Neurological Examination (HINE)<60 and those with HINE≥60

|  |  | HINE<60 (n=16) | HINE≥60 (n=49) | *p* |
| --- | --- | --- | --- | --- |
| Right shoulder | Max | 34.55 (16.41) | 48.20 (24.26) | 0.027**^a^** |
|  | Mean | -0.03 (0.10) | -0.01 (0.18) | 0.710 |
|  | Min | -35.08 (16.39) | -46.72 (22.79) | 0.048**^a^** |
|  | SD | 7.81 (3.65) | 10.83 (4.87) | 0.017**^a^** |
| Left shoulder | Max | 41.13 (32.17) | 47.71 (26.04) | 0.152 |
|  | Mean | -0.03 (0.22) | 0.02 (0.15) | 0.796 |
|  | Min | -39.9 (30.06) | -48.42 (25.06) | 0.120 |
|  | SD | 9.06 (6.69) | 11.32 (5.36) | 0.106 |
| Right elbow | Max | 74.25 (30.79) | 96.11 (31.82) | 0.051 |
|  | Mean | 0.03 (0.28) | 0.02 (0.42) | 0.941 |
|  | Min | -74.4 (35.05) | -93.49 (33.4) | 0.054 |
|  | SD | 17.53 (8.94) | 24.62 (9.31) | 0.017**^a^** |
| Left elbow | Max | 79.59 (37.56) | 93.79 (32.72) | 0.151 |
|  | Mean | 0.17 (0.28) | 0.0 (0.36) | 0.078 |
|  | Min | -76.55 (35.89) | -94.63 (31.19) | 0.057 |
|  | SD | 19.55 (10.76) | 23.95 (9.77) | 0.133 |
| Right hip | Max | 47.1 (26.33) | 57.43 (22.36) | 0.129 |
|  | Mean | -0.14 (0.15) | 0.03 (0.17) | 0.001**^a^** |
|  | Min | -46.95 (27.95) | -55.75 (21.94) | 0.198 |
|  | SD | 10.9 (5.68) | 14.03 (5.60) | 0.080 |
| Left hip | Max | 53.81 (35.37) | 54.85 (21.09) | 0.912 |
|  | Mean | 0.02 (0.12) | 0.05 (0.17) | 0.161 |
|  | Min | -48.59 (28.45) | -49.2 (18.71) | 0.937 |
|  | SD | 10.47 (5.68) | 12.85 (5.23) | 0.196 |
| Right knee | Max | 63.82 (30.12) | 78.72 (29.13) | 0.083 |
|  | Mean | 0.16 (0.31) | -0.05 (0.26) | 0.010**^a^** |
|  | Min | -66.07 (30.16) | -80.37 (29.69) | 0.100 |
|  | SD | 14.97 (8.1) | 20.79 (8.62) | 0.022**^a^** |
| Left knee | Max | 66.31 (35.5) | 72.53 (25.96) | 0.451 |
|  | Mean | 0.04 (0.19) | -0.06 (0.24) | 0.153 |
|  | Min | -67.7 (35.3) | -75.87 (25.39) | 0.316 |
|  | SD | 14.98 (7.78) | 19.46 (7.75) | 0.049**^a^** |

Values are presented as mean (standard deviation).
**^a^***p*<0.05.
