# Supplementary material for: Deep learning-based quantitative analyses of spontaneous movements and their association with early neurological development in preterm infants
Source: Sci Rep. 2022 Feb 24;12:3138. doi: 10.1038/s41598-022-07139-x (PMC8873498; doi:10.1038/s41598-022-07139-x)
Supplement: Supplementary file 3 — Supplementary Information 3. [file 41598_2022_7139_MOESM3_ESM.docx]

**Supplementary Table S3.** Comparison of correlation coefficients for joint angles between preterm infants with Hammersmith Infant Neurological Examination (HINE)<60 and those with HINE≥60

|  | Right  shoulder | Right  elbow | Right  hip | Right  knee | Left shoulder | Left elbow | Left  hip | Left  knee |
| --- | --- | --- | --- | --- | --- | --- | --- | --- |
| Right  shoulder | NA | 0.14 (0.43)  vs  0.06 (0.34), 0.469 | 0.01 (0.23)  vs  -0.05 (0.21),  0.342 | 0.03 (0.22)  vs  0.05 (0.19),  0.831 | 0.22 (0.29)  vs  0.20 (0.21),  0.839 | 0.06 (0.29)  vs  0.02 (0.23),  0.581 | -0.1 (0.21)  vs  -0.07 (0.21),  0.551 | 0.07 (0.24)  vs  0.10 (0.19),  0.682 |
| Right  elbow | 0.14 (0.43)  vs  0.06 (0.34), 0.469 | NA | 0.1 (0.25)  vs  0.03 (0.21),  0.220 | -0.09 (0.24)  vs  -0.01 (0.18),  0.150 | 0.09 (0.20)  vs  0.03 (0.19),  0.314 | 0.21 (0.27)  vs  0.15 (0.25),  0.373 | 0.05 (0.21)  vs  0.03 (0.21),  0.777 | -0.02 (0.22)  vs  -0.04 (0.19),  0.707 |
| Right  hip | 0.01 (0.23)  vs  -0.05 (0.21),  0.342 | 0.1 (0.25)  vs  0.03 (0.21),  0.220 | NA | 0.77 (0.28)  vs  -0.83 (0.11),  0.831 | 0.06 (0.19)  vs  -0.01 (0.18),  0.205 | 0.14 (0.19)  vs  0.11 (0.24),  0.663 | 0.06 (0.35)  vs  0.27 (0.26),  0.016^a^ | -0.22 (0.28)  vs  -0.3 (0.21),  0.238 |
| Right  knee | 0.03 (0.22)  vs  0.05 (0.19),  0.831 | -0.09 (0.24)  vs  -0.01 (0.18),  0.150 | -0.77 (0.28)  vs  -0.83 (0.11),  0.831 | NA | 0.02 (0.23)  vs  0.02 (0.19),  0.446 | -0.15 (0.16)  vs  -0.13 (0.2),  0.769 | -0.21 (0.32)  vs  -0.30 (0.23),  0.484 | 0.29 (0.27)  vs  0.29 (0.22),  0.615 |
| Left shoulder | 0.22 (0.29)  vs  0.20 (0.21),  0.839 | 0.09 (0.2)  vs  0.03 (0.19),  0.314 | 0.06 (0.19)  vs  -0.01 (0.18),  0.205 | 0.02 (0.23)  vs  0.02 (0.19),  0.446 | NA | 0.23 (0.27)  vs  0.08 (0.34),  0.029^a^ | 0.16 (0.23)  vs  0.00 (0.19),  0.010^a^ | 0.08 (0.24) vs  0.02 (0.16),  0.257 |
| Left elbow | 0.06 (0.29)  vs  0.02 (0.23),  0.581 | 0.21 (0.27)  vs  0.15 (0.25),  0.373 | 0.14 (0.19)  vs  0.11 (0.24),  0.663 | -0.15 (0.16)  vs  -0.13 (0.2),  0.769 | 0.23 (0.27)  vs  0.08 (0.34),  0.029^a^ | NA | -0.04 (0.22)  vs  0.06 (0.22),  0.116 | 0.00 (0.2)  vs  -0.04 (0.22),  0.507 |
| Left  hip | -0.1 (0.21)  vs  -0.07 (0.21),  0.551 | 0.05 (0.21)  vs  0.03 (0.21),  0.777 | 0.06 (0.35)  vs  0.27 (0.26),  0.016^a^ | -0.21 (0.32)  vs  -0.3 (0.23),  0.484 | 0.16 (0.23)  vs  0.0 (0.19),  0.010^a^ | -0.04 (0.22)  vs  0.06 (0.22),  0.116 | NA | 0.66 (0.24)  vs  -0.75 (0.24),  0.117 |
| Left  knee | 0.07 (0.24)  vs  0.1 (0.19),  0.682 | 0.02 (0.22)  vs  -0.04 (0.19),  0.707 | -0.22 (0.28)  vs  -0.3 (0.21),  0.238 | 0.29 (0.27)  vs  0.29 (0.22),  0.615 | 0.08 (0.24) vs  0.02 (0.16),  0.257 | 0.00 (0.20)  vs  -0.04 (0.22),  0.507 | -0.66 (0.24)  vs  -0.75 (0.24),  0.117 | NA |

NA: not available.

^a^*p*<0.05.
