# Supplementary material for: Deep learning-based quantitative analyses of spontaneous movements and their association with early neurological development in preterm infants
Source: Sci Rep. 2022 Feb 24;12:3138. doi: 10.1038/s41598-022-07139-x (PMC8873498; doi:10.1038/s41598-022-07139-x)
Supplement: Supplementary file 4 — Supplementary Information 4. [file 41598_2022_7139_MOESM4_ESM.docx]

**Supplementary Table S4.** Comparison of correlation coefficients for joint angular velocities between preterm infants with Hammersmith Infant Neurological Examination (HINE)<60 and those with HINE≥60

|  | Right  shoulder | Right  elbow | Right  hip | Right  knee | Left shoulder | Left elbow | Left  hip | Left  knee |
| --- | --- | --- | --- | --- | --- | --- | --- | --- |
| Right  shoulder | NA | 0.13 (0.38)  vs  0.12 (0.24),  0.896 | -0.04 (0.11)  vs  -0.07 (0.1),  0.278 | 0.05 (0.12)  vs  0.08 (0.1),  0.349 | 0.19 (0.24)  vs  0.17 (0.14),  0.726 | 0.09 (0.17)  vs  0.04 (0.16),  0.361 | -0.05 (0.14)  vs  -0.05 (0.12),  0.920 | -0.03 (0.19)  vs  0.04 (0.11),  0.139 |
| Right  elbow | 0.13 (0.38)  vs  0.12 (0.24),  0.896 | NA | 0.05 (0.15)  vs  0.02 (0.14),  0.472 | -0.02 (0.18)  vs  0.00 (0.13),  0.575 | 0.10 (0.12)  vs  0.07 (0.14),  0.273 | 0.19 (0.18)  vs  0.10 (0.15),  0.128 | -0.03 (0.16)  vs  -0.02 (0.12),  0.916 | 0.04 (0.16) vs  0.00 (0.14),  0.275 |
| Right  hip | -0.04 (0.11)  vs  -0.07 (0.1),  0.278 | 0.05 (0.15)  vs  0.02 (0.14),  0.472 | NA | -0.76 (0.24)  vs  -0.81 (0.13),  0.670 | 0.01 (0.12)  vs  0.00 (0.13),  0.686 | 0.03 (0.13)  vs  0.02 (0.14),  0.819 | 0.18 (0.23)  vs  0.26 (0.22),  0.179 | -0.26 (0.20)  vs  -0.25 (0.2),  0.841 |
| Right  knee | 0.05 (0.12)  vs  0.08 (0.10),  0.349 | -0.02 (0.18)  vs  0.0 (0.13),  0.575 | -0.76 (0.24)  vs  -0.81 (0.13),  0.670 | NA | 0.01 (0.11)  vs  0.00 (0.11),  0.779 | -0.03 (0.12)  vs  -0.04 (0.15),  0.835 | -0.25 (0.20)  vs  -0.26 (0.21),  0.891 | 0.31 (0.17)  vs  0.22 (0.20),  0.114 |
| Left shoulder | 0.19 (0.24)  vs  0.17 (0.14),  0.726 | 0.10 (0.12)  vs  0.07 (0.14),  0.273 | 0.01 (0.12)  vs  0.00 (0.13),  0.686 | 0.01 (0.11)  vs  0.00 (0.11),  0.779 | NA | 0.26 (0.26)  vs  0.12 (0.26),  0.060 | -0.04 (0.15)  vs  -0.02 (0.13),  0.657 | 0.03 (0.13)  vs  0.03 (0.11),  0.901 |
| Left elbow | 0.09 (0.17)  vs  0.04 (0.16),  0.361 | 0.19 (0.18)  vs  0.10 (0.15),  0.128 | 0.03 (0.13)  vs  0.02 (0.14),  0.819 | -0.03 (0.12)  vs  -0.04 (0.15),  0.835 | 0.26 (0.26)  vs  0.12 (0.26),  0.060 | NA | -0.03 (0.13)  vs  0.04 (0.15),  0.110 | 0.06 (0.18)  vs  -0.03 (0.12),  0.057 |
| Left  hip | 0.05 (0.14)  vs  -0.05 (0.12),  0.920 | -0.03 (0.16)  vs  -0.02 (0.12),  0.916 | 0.18 (0.23)  vs  0.26 (0.22),  0.179 | -0.25 (0.20)  vs  -0.26 (0.21),  0.891 | -0.04 (0.15)  vs  -0.02 (0.13),  0.657 | -0.03 (0.13)  vs  0.04 (0.15),  0.110 | NA | -0.66 (0.25)  vs  -0.73 (0.26),  0.196 |
| Left  knee | -0.03 (0.19)  vs  0.04 (0.11),  0.139 | 0.04 (0.16) vs  0.0 (0.14),  0.275 | -0.26 (0.20)  vs  -0.25 (0.20),  0.841 | 0.31 (0.17)  vs  0.22 (0.20),  0.114 | 0.03 (0.13) vs  0.03 (0.11),  0.901 | 0.06 (0.18)  vs  -0.03 (0.12),  0.057 | -0.66 (0.25)  vs  -0.73 (0.26),  0.196 | NA |

NA: not available.
